# The peritoneal tumour microenvironment of high-grade serous ovarian cancer

**DOI:** 10.1002/path.4002

**Published:** 2012-04-18

**Authors:** D Andrew Leinster, Hagen Kulbe, Gemma Everitt, Richard Thompson, Mauro Perretti, Felicity N E Gavins, Dianne Cooper, David Gould, Darren P Ennis, Michelle Lockley, Iain A McNeish, Sussan Nourshargh, Frances R Balkwill

**Affiliations:** 1 Barts Cancer Institute, Barts and The London School of Medicine and Dentistry, Queen Mary University of LondonCharterhouse Square, London EC1M6BQ, UK; 2 William Harvey Research Institute, Barts and The London School of Medicine and Dentistry, Queen Mary University of LondonCharterhouse Square, London EC1M6BQ, UK

**Keywords:** ovarian cancer, peritoneum, metastases, inflammation, chemokines, CXCR4, intravital microscopy

## Abstract

High-grade serous ovarian cancer (HGSC) disseminates early and extensively throughout the peritoneal space, causing multiple lesions that are a major clinical problem. The aim of this study was to investigate the cellular composition of peritoneal tumour deposits in patient biopsies and their evolution in mouse models using immunohistochemistry, intravital microscopy, confocal microscopy, and 3D modelling. Tumour deposits from the omentum of HGSC patients contained a prominent leukocyte infiltrate of CD3^+^ T cells and CD68^+^ macrophages, with occasional neutrophils. Alpha-smooth muscle actin^+^ (α-SMA^+^) pericytes and/or fibroblasts surrounded these well-vascularized tumour deposits. Using the murine bowel mesentery as an accessible mouse peritoneal tissue that could be easily imaged, and two different transplantable models, we found multiple microscopic tumour deposits after i.p. injection of malignant cells. Attachment to the peritoneal surface was rapid (6–48 h) with an extensive CD45^+^ leukocyte infiltrate visible by 48 h. This infiltrate persisted until end point and in the syngeneic murine ID8 model, it primarily consisted of CD3^+^ T lymphocytes and CD68^+^ macrophages with α-SMA^+^ cells also involved from the earliest stages. A majority of tumour deposits developed above existing mesenteric blood vessels, but in avascular spaces new blood vessels tracked towards the tumour deposits by 2–3 weeks in the IGROV-1 xenografts and 6 weeks in the ID8 syngeneic model; a vigorous convoluted blood supply was established by end point. Inhibition of tumour cell cytokine production by stable expression of shRNA to CXCR4 in IGROV-1 cells did not influence the attachment of cells to the mesentery but delayed neovascularization and reduced tumour deposit size. We conclude that the multiple peritoneal tumour deposits found in HGSC patients can be modelled in the mouse. The techniques described here may be useful for assessing treatments that target the disseminated stage of this disease. Copyright © 2012 Pathological Society of Great Britain and Ireland. Published by John Wiley & Sons, Ltd.

## Introduction

There may be controversy about the cell and tissue origin of high-grade serous ovarian cancer (HGSC) 1, 2, but there is no doubt that by the time of diagnosis, this most common and lethal of the ‘ovarian’ malignancies has spread extensively throughout the peritoneal space 3, 4, presenting a fundamental clinical problem. After optimal cytoreduction by surgery and chemotherapy, many microscopic nodules remain, and in these areas chemoresistant cells may develop. The reason why high-grade serous cancers are only detected once they have spread throughout the peritoneum is that small asymptomatic primary lesions, whether they develop on the surface of the ovary or the distal fimbria of the Fallopian tube, are able to generate multiple micro-metastases by the dissemination of malignant cells into the peritoneum 2, 5.

There is as yet no appropriate genetic model of HGSC that accurately recapitulates the p53 mutation and general genomic instability characteristic of this malignancy 6, but intraperitoneal injection of malignant cells in xenogeneic or syngeneic models generates large intraperitoneal tumours and ascites fluid with tumour growth usually measured by bioluminescent imaging of luciferase-transfected cells or weight of macroscopic resected tumours 7–10. The development of microscopic peritoneal metastases has not been studied in detail and it is not known if this is a valid model for the human situation. Our aim was to study the natural history and cellular composition of peritoneal metastases in mouse models and relate our findings to the human disease. We conclude that this clinical problem can be reproduced in mouse models; describe techniques that could enhance our understanding of peritoneal tumour spread and be useful in the development of novel treatments; and show how shRNA to the chemokine receptor CXCR4 had a significant effect on the development of intraperitoneal tumour deposits.

## Materials and methods

### Cell lines

IGROV-1 (NCI) and the ID8 (MOSEC) malignant cells (provided by K Roby, University of Kansas, Kansas City, KS, USA) were used for this study. IGROV-1 cells were cultured in RPMI with 10% FCS and ID8 cells were cultured in DMEM with 4% FCS and insulin transferrin sodium selenite supplement (Sigma Aldrich, Dorset, U.K.). The human cell line most recently underwent 16 loci STR authentication (LGC Standards, London, UK) in September 2011. Both cell lines were infected with a lentiviral eGFP construct (pHRSIN-CSGW-dNotI) to express an eGFP construct (kindly provided by Dr Y Ikeda, Mayo Clinic, Rochester, MN, USA), as described previously by Kulbe *et al* 8. IGROV-1 cells were also transfected with SUPER RNAi™ plasmids containing two different shRNA sequences targeting CXCR4, or a control plasmid containing scrambled RNA (IGROV-Scrambled). Cells were transfected using Lipofectamine 2000 (Invitrogen, Paisley, UK) following the manufacturer's instructions.

### Animal models

ICRF nude mice (Cancer Research UK) were used for the IGROV-1 experiments. C57 Black6 mice (Charles River, Margate, UK) were used for all ID8 experiments. Briefly, IGROV-1 (5 × 10^6^ cells) and ID8 (1 × 10^7^ cells) were injected in 0.3 ml of sterile, endotoxin-free PBS. The animals would reach UKCCCR guidelines for end-point tumour burden 11 in approximately 5 and 12 weeks, respectively.

### Intravital microscopy (IVM)

The protocol for mesenteric IVM has been described previously 12. Body temperature was maintained at 37 °C and mice were anaesthetized with i.p. ketamine (150 mg/kg) and xylazine (7.5 mg/kg). A midline incision (1.5 cm) was made through the skin and the peritoneal wall. The mesentery was positioned over a Perspex viewing stage and observed using a water immersion objective on a Zeiss Axioskop FS microscope (Carl Zeiss, Welwyn Garden City, UK). The number and size of the tumour deposits were recorded in a 0.4 mm^2^ area.

### Fluorescent labelling of the mesentery

Functional blood vessel labelling was achieved by injecting (i.v.) 100 µl of 2 mg/ml lectin solution (TRITC-conjugated *Bandeiraea simplicifolia* lectin; Sigma Aldrich, Dorset, UK) or streptavidin Alexa 633 conjugated to biotin-tagged *Lycopersicon esculentum* lectin (Invitrogen and Vector Labs, Peterborough, UK, respectively) under terminal anaesthesia. The animals were then killed via cardiac perfusion of a 4% paraformaldehyde (PFA) solution. The mesenteric tissue was removed, washed, and fixed in 4% PFA for 1 hour. The tissue was then blocked and permeabilized in a blocking solution of 3% goat serum, 1% BSA, and 1% Triton-X100. Staining for a variety of cell markers was achieved using both fluorophore-conjugated primary antibodies (eg SMA-Cy3 from Sigma; MRP14 conjugated to Alexafluor 555, a kind gift from Professor N Hogg, CRUK; and CD3-Alexa 647 from eBioscience, Hatfield, UK) and primary antibodies with fluorescently labelled secondary antibodies [eg F4/80 (a mouse macrophage-specific marker) with anti-rat DyLight™ 405 from ABD Serotec, Kidlington, UK and CD45 (a common marker for all nucleated haemopoietic cells) from BD, Oxford, UK with anti-rat Alexa 633]. MRP14 was used as a tissue neutrophil marker as it labels calcium-binding proteins highly expressed on neutrophils and monocytes. Tissues were washed in PBS and arranged on a slide. The intestine was then removed before embedding the tissue onto the slide in Prolong Gold (Invitrogen). The mesenteric tissue was imaged on both an Ariol computer-controlled microscope (Leica Microsystems, Gateshead, UK) and image analysis system and a Zeiss LSM 510 confocal microscope.

### Human tumour samples

Human omentum samples were collected from surgery on ovarian cancer patients at St Bartholomew's Hospital. Samples were taken from patients who had given prior consent under the Research Ethical Committee Project number 10/H0304/14 and stored within the Barts Gynae Tissue Bank. Sections of human omental tissue were stained for the immunohistochemical markers CD68 (a marker for all human cells of the macrophage lineage) (Dako, Ely, UK) and CD3 (found on all mature human T cells) (Labvision, Cuncorn, UK) using a Dako Autostainer Plus with a Super Sensitive Polymer—HRP DAB (Launch Diagnistics Ltd, Longfield, U.K.). Neutrophil elastase (a marker for human neutrophils) (Dako) and α-SMA (a marker for fibroblasts and pericytes) (Sigma) staining was visualized with a biotinylated HRP secondary antibody and DAB staining (Vector Labs). Quantification of the tumour-infiltrating inflammatory cells was determined using the Ariol computer-controlled microscope (Leica Microsystems) and image analysis system. Six of the samples were obtained from patients who had interval debulking surgery after three cycles of carboplatin and paclitaxel chemotherapy; two were obtained from patients undergoing primary debulking surgery.

## Results

### Peritoneal metastases of human high-grade serous ovarian cancer

**Figure 1:**
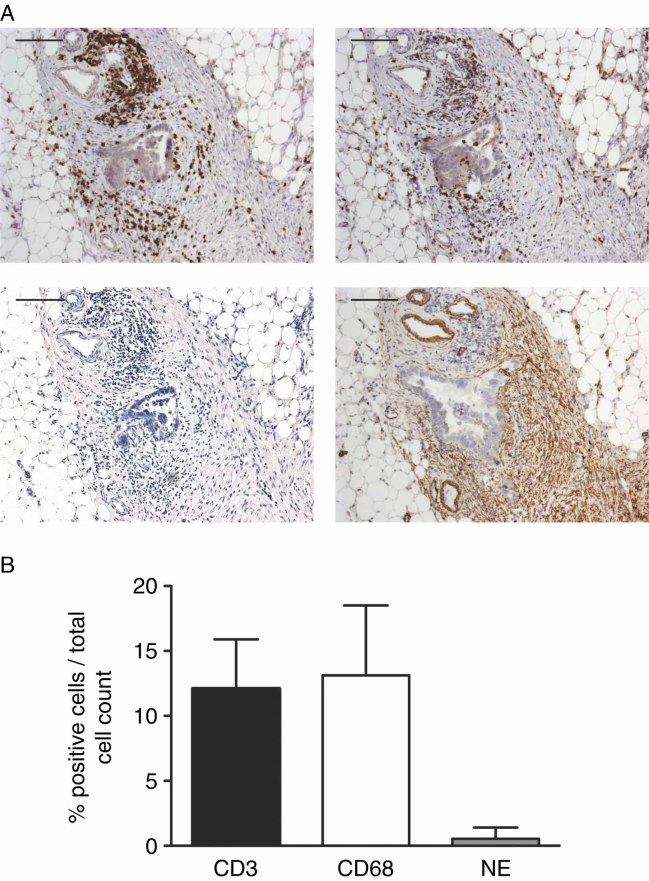
Leukocytes and α-smooth muscle actin (α-SMA)-positive cells in omental tumour deposits of high-grade serous ovarian cancer. Immunohistochemistry was carried out on omental tumour deposits removed at surgery. (A) Representative images of a section from patient G11. Clockwise from top left: CD3^+^, CD68^+^, α-SMA^+^, and neutrophil elastase^+^ (NE^+^) cells. Scale bar = 400 µm. (B) The tumour-infiltrating leukocytes were measured in each entire tissue section using the Ariol computer-controlled microscope and are expressed as a percentage of positively stained cells relative to a haematoxylin nucleus counterstain. All values represent mean and standard deviations. *n* = 8 patients

We first examined the cellular composition of microscopic peritoneal tumour deposits from eight patients with HGSC, using omentum removed during cytoreductive surgery. All deposits had prominent infiltrates of CD3^+^ lymphocytes and CD68^+^ macrophages, but there were few neutrophil elastase-positive cells. The deposits were well-vascularized and had many α-SMA-positive pericytes and fibroblasts (Figure 1A). Using Ariol computer-controlled microscopy, we calculated the percentage of each leukocyte population per total number of cells in the peritoneal tumour islands, assessing an entire tissue section for each of the deposits. Figure 1B shows that 12% of the cells were CD3^+^ T cells and 14% of the cells were CD68^+^ macrophages. Less than 1% of the cells in the tumour deposits were neutrophils.

### Peritoneal metastases of ovarian cancer cells in mice

**Figure 2:**
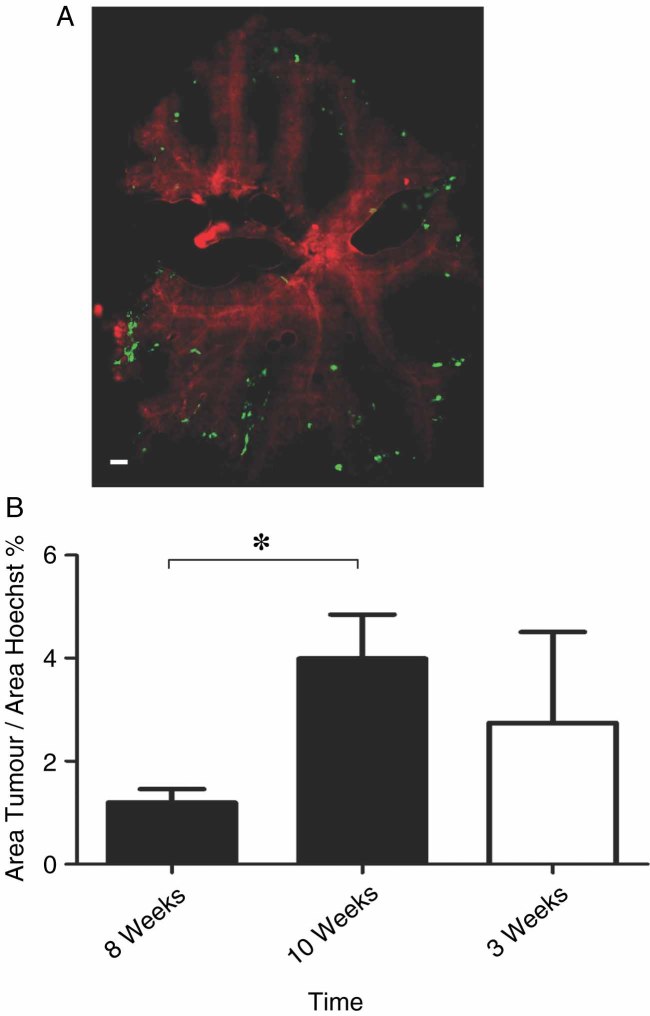
The development of ovarian cancer deposits on the bowel mesentery. Ariol computer-controlled microscopy provided a global view of the colonization of malignant cells on the surface of the mesothelium. (A) A representative image of the colonization of the bowel mesentery with ID8 tumour deposits (green) and the functional vasculature (BS-1 lectin—red). Scale bar = 1 mm. (B) The colonization of the bowel mesentery by tumour cell deposits was measured by the Ariol microscope software by comparing the area of Hoechst staining with the area of the eGFP tumour staining. (Solid bars are the ID8 tumour deposits and the open bars are the IGROV-1 tumour deposits.) The results for the ID8 cells showed a significant increase in the tumour cell area over a 2-week period (*p* = 0.01, Student's *t*-test). *n* = 6 animals per time point for ID8 and *n* = 3 animals for IGROV-1

We used the bowel mesentery as a readily accessible murine peritoneal location for temporal analysis of cellular and molecular changes using both confocal and intravital microscopy. In all experiments, we used two well-characterized transplantable models of ovarian cancer that grow intraperitoneally: IGROV-1 human HGSC xenografts 8, 10 and the ID8 syngeneic mouse ovarian cancer model 7, 9. Both cell lines expressed eGFP. An Ariol computer-controlled microscope imaged the entire bowel mesentery for deposits of eGFP-expressing tumour cells. Vasculature was labelled with red TRITC-labelled *Bandeiraea simplicifolia* lectin. Figure 2A shows a representative image of the bowel mesentery 10 weeks after injection of eGFP-expressing ID8 cells. Similar patterns of spread were observed in a xenograft setting with IGROV-1 cells. A majority of the deposits were located close to existing peritoneal vessels, although some developed in avascular areas. The area of the mesentery that was occupied by eGFP-labelled cells also increased with time, as shown in a typical experiment with ID8 cells (Figure 2B). At 3 weeks, comparable values for IGROV-1 cells were approximately 3% of the mesenteric area (Figure 2B). This reflected the natural history of these two models in our laboratory; the end point of the ID8 model was approximately 12 weeks 9, whereas the IGROV-1 end point was 4–6 weeks 8.

### Intravital microscopy of the tumour-bearing bowel mesentery

**Figure 3:**
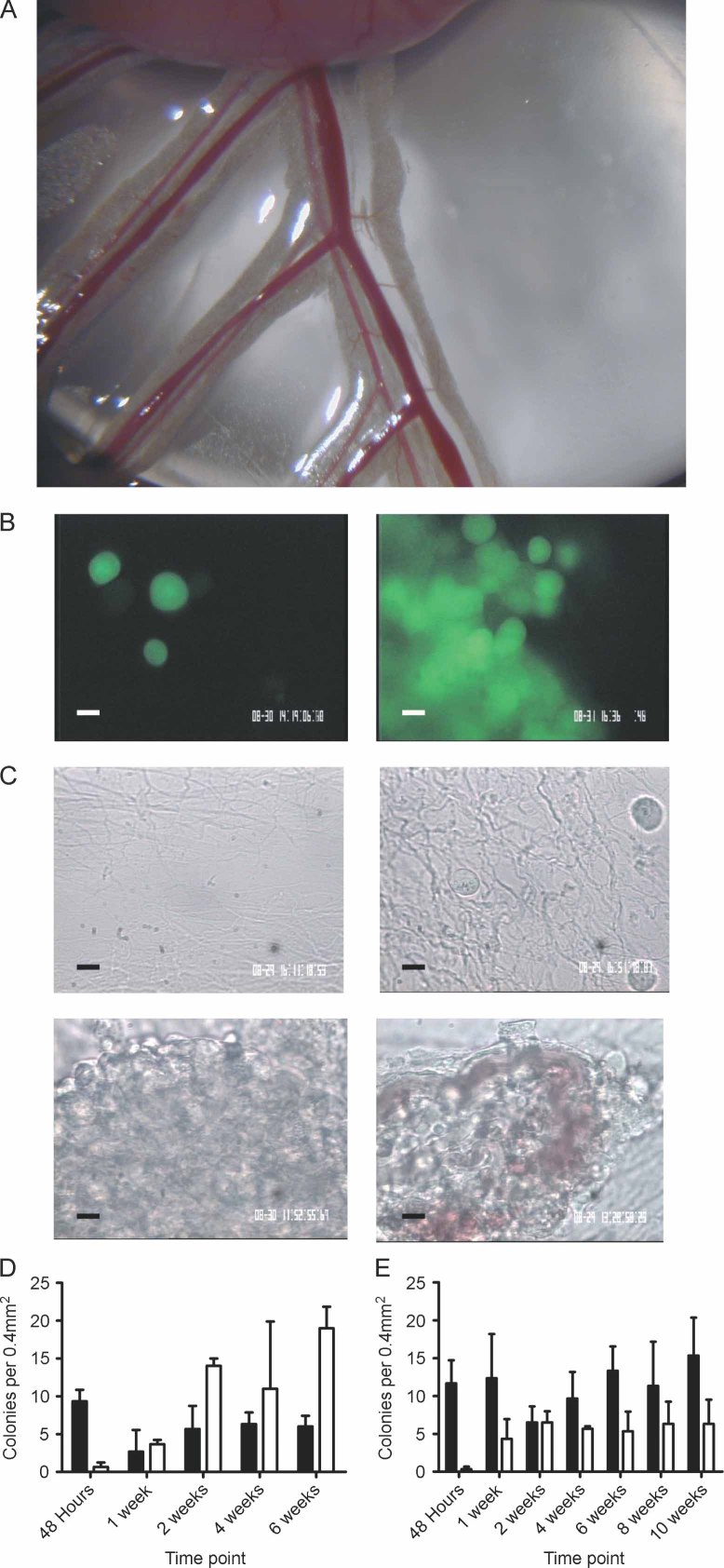
Imaging of tumour deposits on the bowel mesentery by intravital microscopy (IVM). (A) Image of a bowel mesentery preparation as used for the IVM protocols from a dissecting microscope (original magnification × 10). (B) eGFP-transfected malignant cells clearly show the colonization of the bowel mesothelium. Both single cells and larger tumour colonies could be seen. Scale bar = 10 µm. (C) Composite panel showing the development of the IGROV-1 malignant cells on the bowel mesothelium. As mice were sacrificed after imaging, each picture is taken from a different animal. Scale bar = 10 µm. Clockwise from top left: the pre-tumour bowel mesothelium with no malignant cells; the initial colonization process of the malignant cells 48 h post-injection of malignant cells with single cells attached to the mesothelium; the tumour 2 weeks post-i.p. injection with an avascular tumour formed on the surface of the tissue; a vascularized tumour 4 weeks after injection of the cells. (D) Tumour deposits were counted by assessing 40 regions of the bowel mesentery (0.4 mm^2^). The growth of the IGROV-1 malignant cells is shown over a 6-week time course (*n* = 3 animals per time point). (E) ID8 tumour deposits were counted as in D but over a 10-week time course (*n* = 3 per time point). (D, E) Solid bars are colonies smaller than 100 µm in diameter. Open bars are colonies larger than 100 µm

To enable us to study the development of tumour deposits in live animals, an intravital microscopy technique established for studying peritoneal inflammation 12 was used to study peritoneal tumour growth and attachment in more detail. For this purpose, mice were injected with eGFP-labelled malignant cells and at time points between 48 h and 10 weeks, the bowel mesentery was exteriorized under terminal anaesthesia (Figure 3A). Malignant cells were clearly visible in the avascular areas of the mesentery, as observed by both phase contrast and fluorescence microscopy (Figures 3B and 3C). As deposits above and around the existing blood vessels were difficult to image because of surrounding adipose tissue, we focused on areas between the vessels (see Figure 3A).

All such IGROV-1 deposits were avascular for the first 2 weeks. However, by 3 weeks, blood vessels began to spread across the avascular mesenteric spaces towards the malignant cells. At 3 weeks, these vessels had poor flow and were mostly incomplete. After this time, tumour blood vessels developed rapidly and within 6 weeks, most tumours larger than 100 µm in diameter were vascularized with apparently viable structures that could support a fast-moving and convoluted blood flow. (Figure 3C shows this sequence for IGROV-1.) The peritoneal deposits formed by ID8 murine ovarian cancer cells took longer to develop, as expected from our knowledge of the natural history, with new blood vessels developing at 4–6 weeks.

Supplementary Movie 1 (Supporting information) shows the development of IGROV-1 tumours on the bowel mesentery. The normal mesothelium is shown at the beginning, followed by an early time point (6 h post-i.p. injection of cells) where single malignant cells are visible on the mesothelium. After 1 week, the malignant cells showed signs of aggregation. Two weeks after injection, IGROV-1 cells had formed large deposits without a vasculature. The development of a vasculature to the tumour was visible at 3 weeks post-i.p. injection of IGROV-1 cells. The initial vessels appeared as fine structures, and as can be seen in the film, the single red blood cells within them were moving slowly and were sometimes static. By 4 and 6 weeks, the vessels had developed into large structures with an increased flow rate. These images are typical of those obtained from at least three mice at each time point. The images shown at different time points of peritoneal development are from individual mice.

Colony number and size were recorded throughout the experiments. For both the xenogeneic and the syngeneic model, single cells and smaller deposits of less than 100 µm in diameter were visible by 48 h (Figures 3D and 3E). In the IGROV-1 model, the number of small deposits decreased by 1 week and remained constant thereafter. Deposits larger than 100 µm increased in number steadily over the 6-week time course of the experiment. The deposits also grew out from the surface of the bowel mesentery, reaching estimated heights of between 2 and 3 mm at end point. With ID8, the smaller deposits persisted throughout the experiment and larger deposits were observed by 2 weeks. With both models, clusters of cells from the larger deposits could be dislodged, suggesting that they too may be able to disseminate elsewhere through the peritoneal cavity.

### The ‘angiogenic switch’ in the peritoneal tumour islands

**Figure 4:**
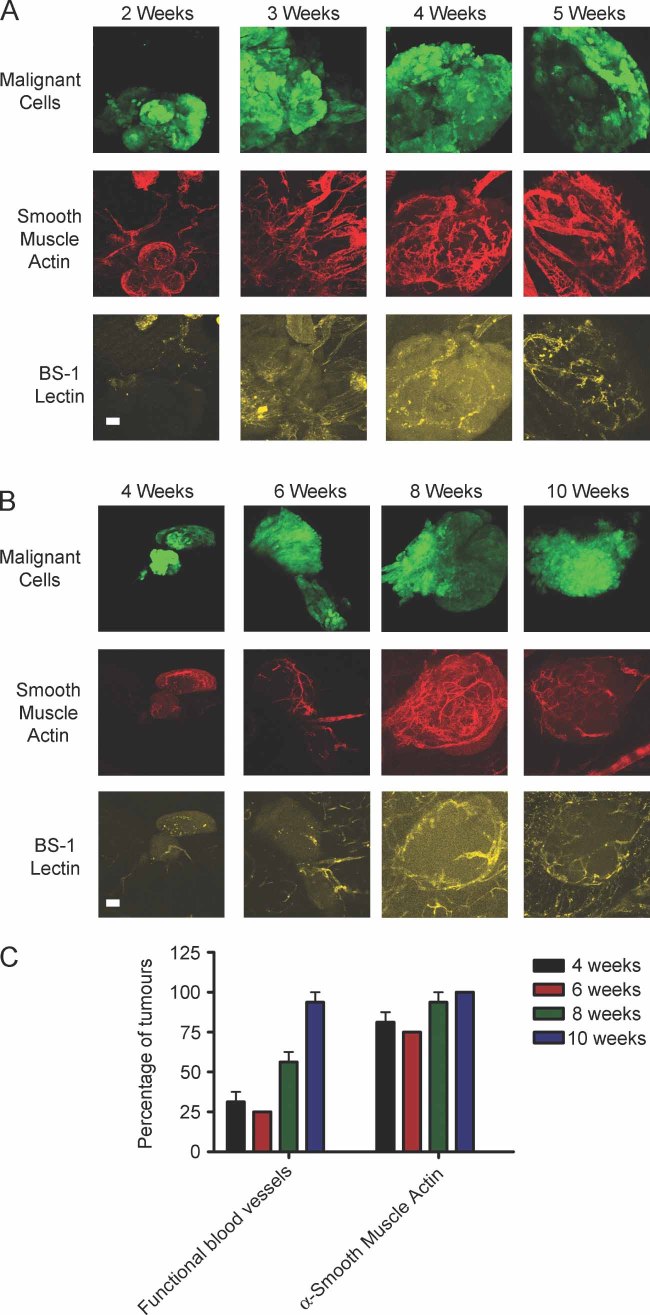
Mesenteric tumour angiogenesis visualized using the whole mount method. (A) The development of IGROV-1 tumours on the bowel mesentery showing the recruitment of functional blood vessels (LE lectin—yellow) and pericytes (anti-SMA—red). Scale bar = 50 µm. (B) ID8 tumours develop over a longer time course compared with the IGROV-1 tumours. However, the similar recruitment of pericytes and functional vessels is visible. Scale bar = 50 µm. (C) Comparison of the pericyte labelling and functional blood vessels in the ID8 tumour model. The percentage of tumours with either BS-1 lectin or anti-SMA labelling is displayed (six tumours analysed per time point)

We next used confocal microscopy to further study the development of blood vessels in the peritoneal deposits, staining the mesothelium for functional blood vessels (*Lycopersicon esculentum* lectin—yellow), fibroblasts, and pericytes (α-SMA—red) with eGFP-positive malignant cells. Figure 4A shows a typical image of IGROV-1 tumour colony development, with vasculature first evident between 3 and 4 weeks. Of note is that α-SMA-positive ‘vascular shapes’ resembling blood vessels were seen before functional blood vessels were observed. In the ID8 model, the vasculature first developed between 4 and 8 weeks post-tumour cell injection (Figure 4B); 25% of tumour deposits were vascularized at 4 weeks but 100% at 10 weeks (Figure 4C). In contrast, the percentage of tumour deposits containing α-SMA-positive cells remained constant over the 4- to 10-week observation period (Figure 4C), suggesting that the pericyte ‘sheaths’ preceded functional blood flow.

### The leukocyte infiltrate in the peritoneal tumour deposits

**Figure 5:**
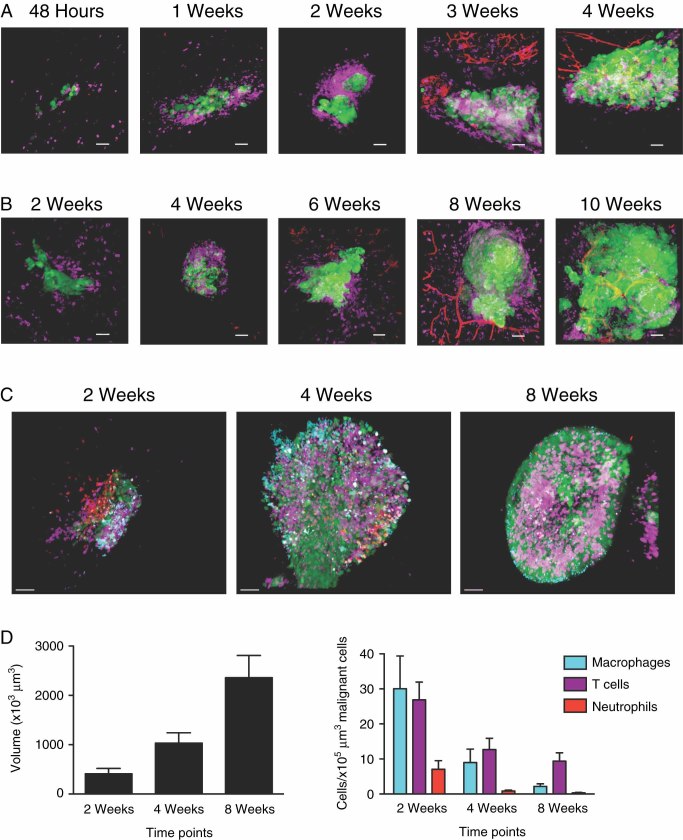
Imaging and quantifying the leukocyte recruitment to mesenteric ovarian tumour deposits. (A) Using immunofluorescence, the IGROV-1 tumours (green) on the bowel mesentery were labelled for immune infiltrate (anti-CD45—purple) and functional blood flow (red). Representative images over a 4-week time course are shown. (B) Using the same labelling as in A, the immune infiltrate and functional blood flow are shown in the ID8 tumour model over a 10-week time period. (C) The different immune cell infiltrate within the ID8 tumour (green) was further characterized by staining for CD3^+^ (purple), F4/80^+^ (turquoise), and MRP14^+^ cells (red) (representative images shown). (D) The tumour volume and immune infiltrate (expressed as number of each cell type per 100000 µm^3^ tumour volume) from the ID8 tumour were quantified using IMARIS analysis software of the CLSM images (18 tumours from six mice). All scale bars = 50 µm. The increase in tumour volume with time was statistically significant (*p* < 0.01, Student's *t*-test). All differences in composition of the leukocyte infiltrate between 2 and 4 or 8 weeks were also significant (*p* < 0.05, Student's *t*-test)

We next examined the leukocyte component of the peritoneal tumour deposits by confocal microscopy using CD45 as a pan-leukocyte marker and red TRITC-labelled lectin as a marker of functional blood vessels. CD45^+^ cells were rarely detected on the bowel mesentery from control mice or in areas of mesentery from tumour-bearing mice that were tumour-free. Within 48 hours of IGROV-1 cell injection, we found leukocytes accumulating around the tumour cell deposits. This leukocyte infiltrate persisted and preceded the development of blood vessels at 3–4 weeks (Figure 5A). As the ID8 model had a slower natural history, we studied this from 2 to 10 weeks after tumour cell injection. Once again, a prominent CD45^+^ leukocyte infiltrate preceded the development of a vasculature to the deposits (Figure 5B). As we had observed with the omental tumour deposits from the HGSC patients, the leukocytes were predominantly CD3^+^ T cells and F4/80^+^ macrophages in the ID8 model (Figures 5C and 5D). There were few MRP14^+^ cells after 2 weeks.

### Manipulation of CXCR4 expression

**Figure 6:**
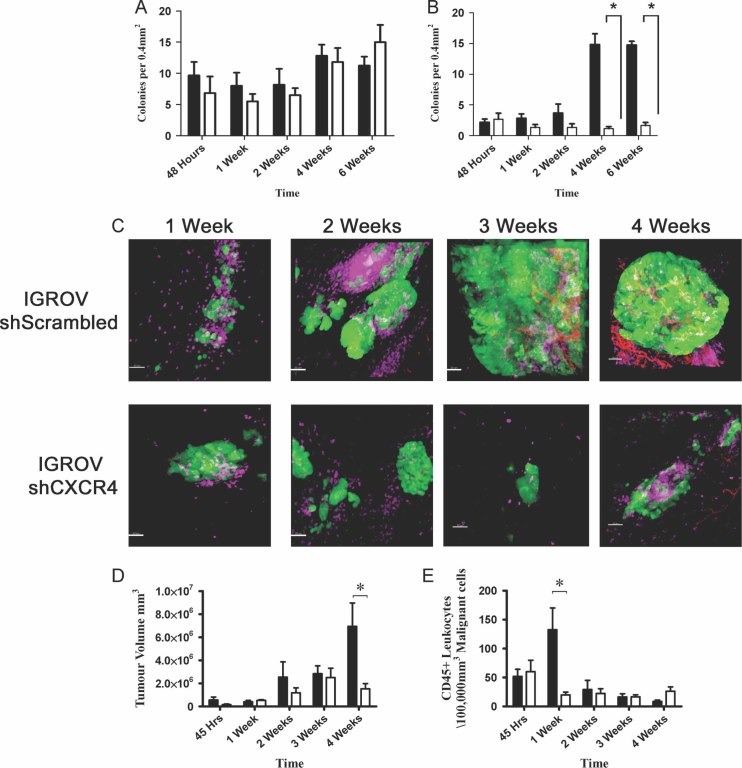
Measuring the effect of CXCR4 shRNAi on the mesenteric development of IGROV-1 ovarian tumours. IVM analysis of the growth of the CXCR4 shRNA IGROV-1 tumours is displayed on the two graphs (black bars—shScrambled IGROV-1 cells; open bars—shCXCR4 IGROV-1).(A) Effect of shCXCR4 on the growth of IGROV-1 cell colonies smaller than 100 µm. (B) Effect of shCXCR4 on the growth of IGROV-1 cell colonies larger than 100 µm. Inhibition of tumour growth was recorded in the larger IGROV-1 deposits, *p* < 0.001 (six mice per time point). (C) Representative images of the leukocyte recruitment (CD45^+^ cells—purple) and functional blood (BS-1 lectin—red) in both mock and shCXCR4 IGROV-1 tumours over a 4-week time period. (D) Measurement of the tumour volume using IMARIS analysis software. *p < 0.001, Bonferroni post-test. (E) The density of CD45^+^ leukocytes. *p < 0.001, Bonferroni post-test). All scale bars = 50 µm

The results described above show that we can model the development of peritoneal deposits of ovarian cancer in mice and that the leukocyte populations resemble those seen in human HGSC. We therefore asked if we could use our methods to measure changes in tumour development. We chose to assess the effects of manipulating expression of the chemokine receptor CXCR4 on peritoneal tumour deposits, as we, and others, have found this receptor to be important in ovarian cancer growth and spread 8, 13–15. Moreover, we recently reported that stable knockdown of CXCR4 reduced tumour growth *in vivo*, as measured by imaging of luciferase-transfected cells 10. The efficacy of the CXCR4 knockdown and the lack of effect on *in vitro* growth and viability of these cells have already been published by us 10. IGROV-1 cells mock-transfected or transfected with shRNA to CXCR4 were injected i.p. and peritoneal tumour colony formation was assessed over a 6-week time period using intravital and confocal microscopy. Intravital microscopy showed that more mock-transfected than shCXCR4 tumour cells had adhered to the mesentery in the time period 48 h–2 weeks. Control deposits developed and grew in size as expected. Viable single cells and small deposits remained on the mesentery in the shCXCR4 group but the number of deposits 100 µm in diameter or more was greatly reduced (*p* < 0.001) (Figures 6A and 6B). Confocal microscopy revealed a diminished vasculature in the shCXCR4 deposits (Figure 6C). We also used IMARIS analysis to measure tumour volume and CD45^+^ leukocyte density. At 4 weeks, there was a significant decrease in tumour volume and at 1 week a significant decrease in leukocyte count standardized to tumour volume (Figures 6D and 6E) (both *p* < 0.001).

## Discussion

Traditional concepts of metastasis may not apply to some types of ovarian cancer. In HGSC, there is no anatomical barrier to seeding throughout the peritoneal cavity. At the earliest detectable stage, the disease may have already formed multiple small peritoneal deposits and these can probably be generated from a primary cancer no larger than a few centimetres 2, 5. Any therapeutic approach to this disease therefore needs to take into account an understanding of the development of peritoneal deposits and their response to treatment. Here we have shown that in experimental mouse models, ovarian cancer cells injected intraperitoneally attach rapidly to avascular areas of the peritoneal surface, forming vascularized deposits. These tumour deposits contain substantial infiltrates of lymphocytes, macrophages, and fibroblasts/pericytes that mimic the composition of those found in patients with advanced HGSC. After i.p. injection, the malignant cells also formed deposits over the vascular areas of the bowel mesentery and some of these may have been associated with milky spots as described by Gerber *et al* 16. However, we conclude that, at least on the bowel mesentery, malignant cells can attach to mesothelial cell monolayers and then recruit their own microenvironment of leukocytes and new blood vessels.

The infiltrating leukocytes found in primary human HGSC tumours are related to both good and bad prognosis, depending on subtype. Increased infiltration of CD8^+^ lymphocytes in solid tumour islets predicts longer survival 17 and correlates significantly with BRCA loss 18. On the other hand, high numbers of CD4^+^ T-regulatory cells, which can mediate immune suppression, predict poor patient survival 19, 20. Additional immunosuppressive leukocyte subtypes, such as B7-H4-expressing tumour macrophages 21, have also been correlated with poor outcome in ovarian cancer.

Six of the eight patients studied had already received three cycles of chemotherapy. As a majority of HGSC patients in the UK are now pretreated before surgery, we could only obtain two chemo-naïve samples. We could see no difference between untreated and treated in terms of deposit size, location or the CD3^+^ and NE^+^ infiltrate but there did seem to be fewer CD68^+^ cells in the treated patients.

We believe that any pre-clinical studies of HGSC in mouse models should study peritoneal spread, as this is the major clinical challenge. There is evidence for a cancer stem cell (CSC) population in HGSC with the capacity for both self-renewal and generating heterogeneous lineages 22–25, and we expect that this stem cell component will be a major determinant of cell behaviour *in vivo* 23. It is likely that the tumour deposits that have colonized the peritoneum in the mouse models are derived from these CSCs. We have shown that in HGSC there is an autocrine cytokine network in the malignant cells, with the major players being CXCR4, CXCL12, TNF-α, and IL-6. We have named this the TNF network. This network has paracrine tumour-promoting actions on the leukocyte infiltrate and angiogenesis 10. The results presented here show that inhibiting the TNF network via stable CXCR4 knockdown can have profound effects on the development of peritoneal deposits. Viable malignant cells survived attached to the peritoneal surface but few developed any vasculature. Treatment of HGSC with cytokine or chemokine antagonists, eg anti-IL-6 antibodies 26, may be one way to delay or inhibit further development of peritoneal disease after diagnosis and initial chemotherapy and surgery.

## SUPPORTING INFORMATION ON THE INTERNET

The following supporting information may be found in the online version of this article.

**Supplementary Movie 1.** Intravital microscopy of the bowel mesentery showing the development of peritoneal tumour deposits.

## Abbreviations

BS-1: *Bandeiraea simplicifolia*
HGSC: high-grade serous ovarian cancer
i.p.: intraperitoneal
i.v.: intravenous
IVM: intravital microscopy
LE: *Lycopersicon esculentum*
NE: neutrophil elastase
