# Supplementary material for: The peritoneal tumour microenvironment of high-grade serous ovarian cancer
Source: J Pathol. 2012 Apr 18;227(2):136–45. doi: 10.1002/path.4002 (PMC3609073; doi:10.1002/path.4002)
Supplement: Supplementary file 1 [file path0227-0136-SD1.docx]

**Supporting information**

**Supplementary movie legend**

**Supplementary Movie 1. Intravital microscopy of the bowel mesentery showing the development of peritoneal tumour deposits**

The normal mesothelium is shown at the beginning, followed by an early time point (6 h post i.p. injection of cells) where single malignant cells are visible on the mesothelium. After 1 week, the malignant cells showed signs of aggregation. Two weeks after injection, IGROV-1 cells have formed large deposits without a vasculature. The development of a vasculature to the tumour was visible at 3 weeks post i.p. injection of IGROV-1 cells. The initial vessels appeared as fine structures, and as can be seen in the film, the single red blood cells within them were moving slowly and were sometimes static. By 4 and 6 weeks, the vessels have developed into large structures with an increased flow rate. These images are typical of those obtained from at least three mice at each time point. The images shown at different time points of peritoneal development are from individual mice.
